# Molecular Mechanisms of Coffee on Prostate Cancer Prevention

**DOI:** 10.1155/2022/3254420

**Published:** 2022-04-22

**Authors:** Julia Montenegro, Otniel Freitas-Silva, Anderson Junger Teodoro

**Affiliations:** ^1^Laboratory of Functional Foods, Program of Food and Nutrition, Federal University of the State of Rio de Janeiro, UNIRIO, Rio de Janeiro, RJ, Brazil; ^2^Department of Agricultural, Food and Nutritional Science, University of Alberta, Edmonton, Alberta, Canada; ^3^Brazilian Agricultural Research Corporation, Embrapa Food Agroindustry, Rio de Janeiro, RJ, Brazil

## Abstract

Prostate cancer (PCa) is one of the most common types of cancer among men, and coffee is associated with a reduced risk of developing PCa. Therefore, we aim to review possible coffee molecular mechanisms that contribute to PCa prevention. Coffee has an important antioxidant capacity that reduces oxidative stress, leading to a reduced mutation in cells. Beyond direct antioxidant activity, coffee stimulates phase II enzymatic activity, which is related to the detoxification of reactive metabolites. The anti-inflammatory effects of coffee reduce tissue damage related to PCa development. Coffee induces autophagy, regulates the NF-*κ*B pathway, and reduces the expression of iNOS and inflammatory mediators, such as TNF-*α*, IL-6, IL-8, and CRP. Also, coffee modulates transcriptional factors and pathways. It has been shown that coffee increases testosterone and reduces sex hormone-binding globulin, estrogen, and prostate-specific antigen. Coffee also enhances insulin resistance and glucose metabolism. All these effects may contribute to protection against PCa development.

## 1. Introduction

The prostate is a gland localized between men's bladder and penis and surrounds the urethra. It secretes prostate fluid that protects sperm. Prostate cancer (PCa) develops when mutated semen-secreting prostate cells start proliferating uncontrollably. If PCa is not treated, it may metastasize, usually to lymph nodes, to hip bone, and then to other organs. Ordinarily, it is not diagnosed in the early stages because it is initially asymptomatic [1]. PCa is the second most common type of cancer among males, accounting for 14.1% of all cancers in men (WHO 2020).

Cancer, in general, develops due to successive mutations in genes, which alter cell morphology and physiology [2]. Oxidative stress leads to inflammation through redox pathways, increasing inflammatory marker circulation [3, 4]. The inflammatory response causes tissue injury and DNA damage [3, 5]. This disorder affects cell cycle and cell division, apoptosis signaling, and DNA repair mechanisms, leading to cancer [2].

Besides nonmodifiable risk factors, such as age, family history, and ethnicity, PCa development can also be influenced by diet and environmental factors, through epigenetics, which involves changes of gene transcription without any alteration in the nucleotide sequence [1]. Consumption of high content of natural phytochemicals from plants is associated with health benefits [4].

Coffee can be a major source of biochemical compounds [6] that can contribute to protection against PCa. In 2020, coffee consumption worldwide was 167.2 million bags (60 kg/bag) ((ICO) 2021). The main varieties produced are Arabica (*Coffea arabica*, 58.5%) and Robusta (*Coffea canephora*, 41.5%) ((ICO) 2021). Coffee has shown *in vitro* antiproliferative effects against PCa cell lines [7–11].

There are some meta-analyses with epidemiological data exploring the association of coffee intake and PCa risk [12–17]. However, no reviews so far have explored how coffee can have those beneficial effects on PCa. Therefore, the aim of this review is to detail molecular mechanisms that can be involved in the capacity of coffee and its bioactive compounds to prevent and treat PCa.

## 2. Methods

In this review, we searched at Web of Science, Scopus, and PubMed databases. The search terms included “Coffee” OR “*Coffea arabica*” OR “*Coffea canephora*” OR “chlorogenic acids” OR “CGA” OR “caffeine” on the title or abstract. Those were combined with specific terms of each possible effect with the Boolean operator “AND” for each search. For the antioxidant, the terms were “anti-oxidant” OR “oxidative stress” OR “Reactive oxygen species”. For the anti-inflammatory, the terms were “anti-inflammatory” OR “inflammation” OR “cytokines”. Other terms were “mutation”, OR “DNA damage” OR “transcriptional factors” OR “signaling pathways” OR “testosterone” OR “steroids hormones” OR “insulin resistance”.

Papers retrieved were filtered for original articles, published in English in the last 10 years. The association between coffee intake and prostate cancer risk has sometimes produced inconsistent results. To circumvent, summarize, and assess the quality of current evidence on the subject, observational studies on molecular mechanisms on existing findings were considered. Only papers produced in high impact factor journals were considered and used in this research. It was also observed the heterogeneity, evidence of small-study effects, and excess significance bias.  Table 1 summarizes the relevant findings from 38 articles included in the present review.

## 3. Bioactive Compounds in Coffee

Coffee contains many well-known bioactive compounds that can be related to anticancer effects. Caffeine is a trimethylxanthine and is the major bioactive compound in coffee [18]. Virtually, all caffeine is absorbed and its main effect is the stimulation in the central nervous system and adenosine receptor inhibition [18, 19]. It has been observed that caffeine increases cancer cell death and protects against mutagenicity [20].

Chlorogenic acids (CGAs) are a group of polyphenols formed between transcinnamic and quinic acid [19, 21]. CGAs prevent free radical damage and modulate inflammation, regulation of glucose, and lipid metabolism [20]. Trigonelline is generated from the nicotinic acid and regulates key enzymes in glucose and lipid metabolism, preventing cell invasion and inhibiting cancer cell proliferation [18]. Diterpenes are the main lipidic fraction in coffee, primarily cafestol and kahweol, and have anticarcinogenic, antioxidant, and anti-inflammatory activity [18, 20]. Maillard reaction forms melanoidins during roasting, which has antioxidant, anti-inflammatory, and antimicrobial effects [18].

However, the chemical composition of coffee can vary depending on various factors. The first factor is the variety. Robusta coffee has more chlorogenic acids and caffeine than Arabica [22]. During coffee farming, the soil, altitude, sun exposure, rain, and temperature can affect coffee composition [23]. During processing, roasting, grinding, and brewing also affect the final composition [10].

## 4. Antioxidant Activity Induced by Coffee

Reactive oxygen species (ROS) and reactive nitrogen species (RNS) are produced endogenously by mitochondrial respiration and exogenously by exposure to oxidizing agents [24]. Oxidative stress results from cellular production of oxidant molecules surpassing the capacity of antioxidants to overcome these damages and may lead to PCa development [25]. Oxidative stress causes the oxidation of crucial biomolecules, causing DNA damage and oxidizing key enzymes involved in gene expression [24].

**Figure 1:**
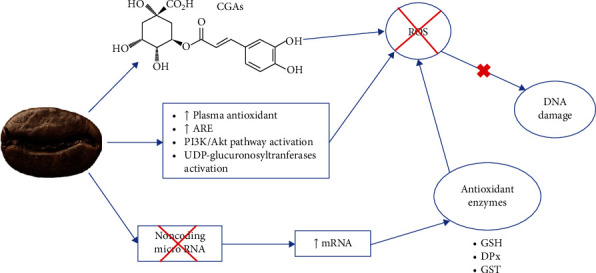
Mechanism of coffee's antioxidant activity.

Coffee presents high antioxidant capacity *in vitro* [10, 26, 27]. Coffee's antioxidant compounds include caffeine, phenolic compounds (mostly CGAs), trigonelline, diterpenes (cafestol and kahweol), and melanoidins [20, 26]. A mechanism for coffee's antioxidant activity is described in Figure 1.

Some studies have reported a significant increase in total plasma antioxidant capacity following coffee consumption, even after a single serving (200 or 400 mL). These effects of a single dose were lost in long term, but in recurrent consumption of medium roast coffee (150 mL/day), plasma antioxidant activity was increased by up to 26% [25].

Coffee significantly increased antioxidant response element (ARE) activation, which could induce the expression of genes related to the cellular antioxidant system [28, 29]. ARE proteins are a part of the complex antioxidant system that protects cells from oxidative damage by neutralizing free radicals and oxidizing agents [30]. ARE-related genes are in the cell defense promoter regions, which include phase II detoxifying enzymes and enzymes involved in antioxidant defense [29, 31].

Coffee consumption increases the intracellular activity of phase I (cytochrome P450) and II enzymes [6], such as glutathione reductase (GR), reduced glutathione (GSH), glutathione peroxidase (GPx), glutathione S-transferases (GST), superoxide dismutase (SOD), and catalase (CAT) ([32], Valadão [29, 30, 33–36]). This increase in GSH has been attributed to polyphenols [34].

It has been hypothesized that compounds with antioxidant properties generally increase the messenger RNA (mRNA) expression of antioxidant-related enzymes. But the expression of those enzymes can be downregulated in some antioxidant-treated cells because the compounds may have directly ameliorated the prevailing oxidative stress [34].

A low-molecular-weight coffee fraction supplementation in rats was able to reduce noncoding microRNA-124-3p and increase the expression of mRNA involved in GPx coding, raising the expression of this enzyme, probably due to caffeine [37]. Noncoding microRNA is an epigenetic factor, can act as tumor suppressors or oncogenes, and may be downregulated or upregulated in PCa. They are short regulatory RNA molecules that cannot be translated into amino acids and may disturb the mRNA purpose. This might influence RNA silencing and gene expression at posttranscriptional and translational levels [1].

Coffee has also been shown to inhibit oxidative stress through UDP-glucuronosyltransferases (UGT) activation, which catalyze the detoxification of reactive metabolites [38]; similar results were observed for caffeic acid [39]. Coffee reduced ROS production/concentration [38, 40, 41].

Antioxidant properties observed in coffee are mainly attributed to CGAs, which have one to two aromatic rings linked to hydroxyl groups and donate hydrogen atoms, reducing free radicals [24, 40, 42, 43]. Their oxidation products, phenoxyl radicals, are promptly stabilized by resonance stabilization. CGAs react with different sources of free radicals at a varied pace; their relative efficiency is species-specific [24].

Isolated kahweol protected mitochondria from redox stress and prevented the formation of ROS and RNS (De Oliveira, De Souza, and Fürstenau [44]).

## 5. Anti-Inflammation Effects

Inflammation is a physiological reaction to tissue damage induced by exogenous or endogenous agents. Exogen factors include pathogens, allergens, foreign bodies, and pernicious substances. Endogenous causes originate from cell signaling due to injured or malfunctioning tissues [24].

Inflammation may be related to PCa development because inflammatory cells are often present in the prostate microenvironment of adult men and are related to PCa precursor lesions, called proliferative inflammatory atrophy, which is abundant in cells that may be predisposed to genomic mutations, and inflammatory stress can provoke epigenetic changes, concomitant with the rupture of the epithelial barrier [45].

Coffee compounds can decrease chronic inflammation and, therefore, protect against DNA degradation, consequently decreasing the risk of disease [46]. The inflammation process is characterized by the raised production of proinflammatory cytokines, such as C-reactive protein (CRP), interleukins (IL), and tumor necrosis factors (TNF) [34].

**Figure 2:**
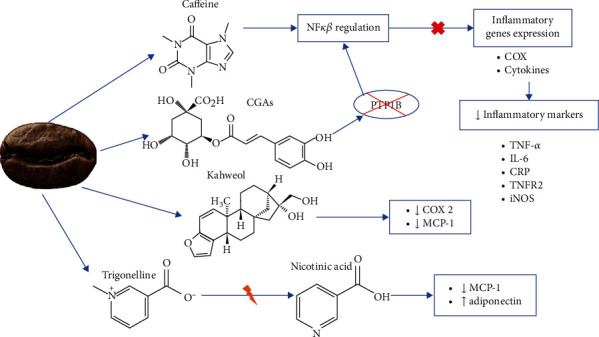
Mechanism of action of coffee for anti-inflammatory activity.

A proposed mechanism for coffee's anti-inflammatory effects is described in Figure 2. In animal models, green coffee intake reduced inflammatory markers, such as TNF-*α* [47, 48], IL-6, IL-10 [48], IL-1*β*, and Nos2 and reduced interstitial inflammatory index [47]. It has been observed in clinical trials that coffee intake over several weeks had a prevailing anti-inflammatory action evaluated by serum markers [49]. In addition to blood inflammatory markers, topical treatment of coffee and caffeine on mice's paws displayed a considerable inhibition of the carrageenan-induced oedema development [27].

It has been observed that coffee can inhibit TNF-*α*-induced NF-*κ*B activity and DNA-binding in PCa cells. Coffee also regulated the expression of inflammatory and cancer-related genes probably through the NF-*κ*B signaling pathway. Coffee downregulated genes related to invasion (MMP9) and inflammation (NF-*κ*B2, CD40, EDN1, and ICAM1) and upregulated genes related to the antioxidant system, such as NFE2L2, HMOX1, NQO1, and GCLC [8].

The TNF-*α* and IL-6 expressions were decreased by lightly roasted coffee extract, and the levels raised as roasting levels were exacerbated [34]. Inducible nitric oxide synthase (iNOS) expression declined, which produces proinflammatory mediators, such as NO [26, 34]. Coffee exhibited an inverse relation with CRP, IL-6, and TNFR2, with similar results both in caffeinated and decaffeinated coffee [50].

Autophagy selectively degrades cellular components to minimize cell injury [3]. Autophagy is related to inflammation and immunity regulation and has tumor-suppressive properties [51]. It has been observed that caffeinated and decaffeinated coffee can induce autophagy. Chronic and acute administration of coffee raised the autophagic flux in different tissues and reduced the content of the autophagic substrate sequestosome-1 (p62/SQSTM1). This occurs through lipidation of microtubule-associated protein 1 light chain 3 *β* (LC3B-I), rising the electrophoretic mobility in sodium dodecyl sulfate PAGE (SDS-PAGE), and converting to LC3B-II. Coffee also caused inhibition of the mammalian target of rapamycin complex 1 (mTORC1) enzymatic activity, probably related to deacetylation of cellular proteins [52]. Caffeine is known to induce hepatic autophagy, being hepatoprotective ([53, 54], Salomone, Galvano, and Li Volti [55]).

Indeed, caffeine is the main compound with overall anti-inflammatory activity in coffee and can reduce TNF-*α* and IL-6 production in a dose-dependent way. Caffeine can modulate nuclear factor-*κ*B (NF-*κ*B) activation [28], which provokes the expression of inflammatory genes, including iNOS, cyclooxygenase-2 (COX2), and cytokines [34].

Nonetheless, 26 anti-inflammatory metabolites have been identified in coffee [48]. CGAs decrease the generation of inflammatory mediators by inhibition of protein tyrosine phosphatase 1B (PTP1B), minimizing proinflammatory cytokine genes expression and regulating NF-*κ*B activation [56]. As a result, COX is suppressed, causing a reduction of IL-6 and IL-8 and TNF-*α* release [28, 34]. Great CGA concentration represses IL-1B mRNA, provoking considerably less cell adhesion and inflammation [46].

Kahweol lessens COX2 and monocyte chemoattractant protein-1 (MCP-1) quantities, meaning it could be antiangiogenic. Kahweol also decreases iNOS in rats' carrageenan paw oedema. During roasting, trigonelline is fractionated in nicotinic acid, which is a promising anti-inflammatory agent, as it diminishes MCP-1 and enhances adiponectin in adipocytes infused with TNF-*α* [46].

## 6. Protection against DNA Damage

**Figure 3:**
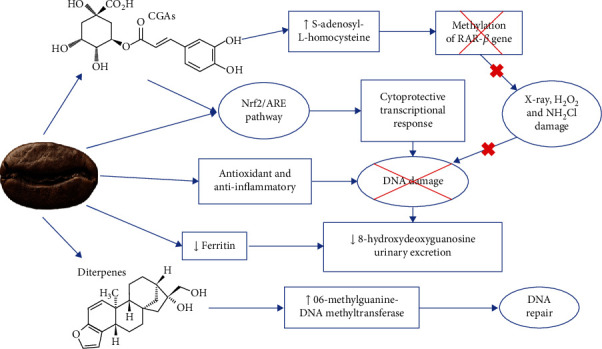
Mechanism of action of coffee for protection against DNA damage.

Coffee's antioxidant and anti-inflammatory effects result in protection against DNA damage. A proposed mechanism for coffee protection against DNA damage is described in Figure 3. It has been observed a reduction in spontaneous DNA strand breaks after only 2 hours of coffee consumption, with further decrease when more coffee was consumed [57]. Similar results were observed in 4 weeks [32, 58] and 8 weeks [31] of coffee intake.

These observations imply a defensive impact of coffee on DNA integrity. It has been observed that coffee reduced oxidative DNA damage induced by hydroxyl (OH°) radicals [43], H_2_O_2_ [33, 41], Ro photosensitizer [41], and benzo[*α*]pyrene (BaP) [38]. Additionally, coffee presents strong chemopreventive properties against DNA damage caused by aflatoxin, probably due to induction of GST [59].

Isolated compounds found in coffee, such as CGA and caffeine metabolites, reduced DNA single-strand breaks caused by ROS [60]. CGA isomers displayed a protective action against X-ray, H_2_O_2_, and NH_2_Cl-induced DNA plasmid chromosome breaks [24].

Urinary 8-hydroxydeoxyguanosine excretion tended to reduce with coffee intake, which is a biomarker of systemic oxidative DNA damage and repair. This result was associated with lower serum ferritin and, indeed, coffee has iron-chelating properties [61]. Coffee and its diterpene components kahweol and cafestol raised the expression of DNA repair protein O6-methylguanine-DNA methyltransferase and some phase II enzymes [57].

Coffee constituents, such as CGAs, trigonelline, and kahweol, are regulators of the Nrf2/ARE signaling pathways ([57, 62], De Oliveira, De Souza, and Fürstenau 2020). It has been observed raised levels of Nrf2-dependent enzymes after consumption of coffees rich in CGA. Likewise, an inverse correlation between Nrf2 transcription and DNA strand breaks has been observed after coffee consumption [58].

Both Nrf2 and phosphorylated Nrf2 (pNrf2) are involved in the induction of Nrf2/ARE-dependent gene transcription. Alterations in Nrf2 translocation mediate the ARE-linked cytoprotective transcriptional response rather than a change in total Nrf2 concentration. There is evidence of pNrf2 translocation to the nucleus and an increase in pNrf2 with the decrease in Nrf2 due to coffee consumption [29, 31, 62].

## 7. Modulation of Transcriptional Factors

**Figure 4:**
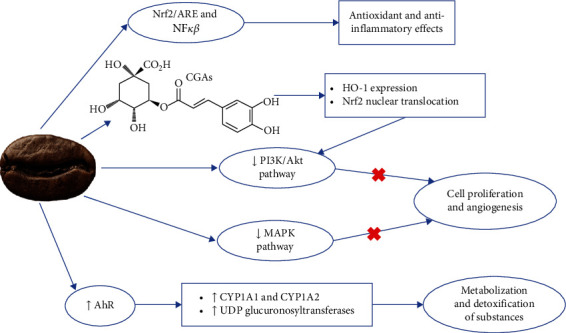
Mechanism of action of coffee for modulation of transcriptional factors.

Coffee may prevent PCa development through modulation of transcriptional factors, which mechanism is shown in Figure 4. Some of those factors have already been discussed, such as NF-*κ*B and Nrf2/ARE pathways. Coffee may also affect phosphatidylinositol-3-kinase/protein kinase B (PI3K/Akt), activator protein 1 (AP-1), aryl hydrocarbon receptor (AhR), and mitogen-activated protein kinase (MAPK) pathways.

Coffee can downregulate PI3K/Akt signaling pathway [56], as well as its compounds, like caffeine [63] caffeic acid, and kahweol (De Oliveira, De Souza, and Fürstenau [44]). This pathway is protooncogenic and is responsible for metabolism, cell cycle, survival, and angiogenesis. It is frequently engaged in heme-oxygenase 1 (HO-1) expression and transcription of several kinds of cells. CGA promotes HO-1 expression and Nrf2 nuclear translocation, which might be related to PI3K/Akt signaling pathway [33].

The MAPK group comprises extracellular responsive kinase (ERK), c-Jun N-terminal kinase (JNK), and p38 MAPK. MAPK signaling pathways are regulated by coffee [56] and kahweol (De Oliveira, De Souza, and Fürstenau [44]). There is an association between the restriction of cell growth and the lesser activation/phosphorylation of MAPKs, indicating a capacity to decrease cancer cell proliferation [64].

AhR has an adjusting role in the manifestation of CYP1A1 and CYP1A2 genes, which are involved in the metabolization of several substances [65]. Coffee activates AhR pathways genes, inducing UGT, which is related to detoxification and clearance of reactive metabolites [38, 66].

There is evidence that CGA secures against cancer caused by external factors. Its defensive actions can be associated with the omission of NF-*κ*B, AP-1, and MAPK activation concerning ROS effects [64]. Specific effects of CGAs in transcriptional pathways leading to cancer growth suppression are reviewed elsewhere [21].

## 8. Controlling Steroid Metabolism

Historically, it was believed that androgens were involved in PCa development. However, more recent studies found no relation between testosterone levels and/or testosterone therapy and increased risk of PCa [67–73]. Furthermore, androgen deprivation therapy (ADT) is effective against most types of PCa, but its effect is probably related to androgen receptor (AR) expression, which is a well-established component of PCa [74–76].

**Figure 5:**
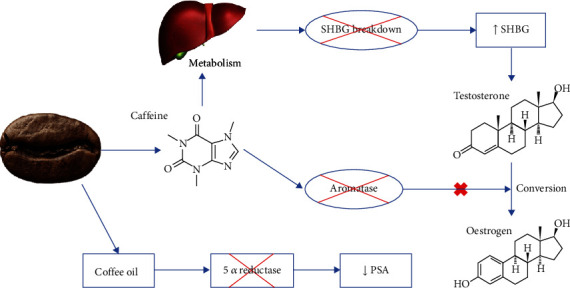
Mechanism of action of coffee for controlling steroid metabolism.

Therefore, the relationship between androgenic hormones and PCa development is not completely understood. But coffee is involved in steroid metabolism, and a proposed mechanism is described in Figure 5.

Caffeinated coffee is associated with higher concentrations of total testosterone. This is probably related to sex hormone-binding globulin (SHBG), which is a sex hormone transport protein and functions as a regulator of their activity. Decreased SHBG may be linked to an elevated risk of PCa [50]. Coffee and caffeine are linked to greater SHBG concentration [50, 77, 78]. Caffeine and SHBG are primarily metabolized by the liver, so one possible explanation is that caffeine intake might cause SHBG rise by impacting its catabolism [50]. Another possibility is that caffeine increases hepatic SHBG production by upregulation adiponectin synthesis and decreasing Akt phosphorylation [77].

Besides that, studies have shown that coffee and caffeine can affect estrogen metabolism by inhibiting aromatase, the prime enzyme responsible for the transformation of androgen to estrogen [50, 79]. Serum estrogen and estrogen receptors (ER) are associated with PCa development, and the molecular mechanisms involved are reviewed elsewhere [80, 81]. Coffee intake is related to reduced levels of estrogen [50], possibly related to the presence of compounds with estrogenic activity in coffee [82]. Caffeine and caffeic acid reduced the ER expression [63].

Coffee oil has significantly decreased the prostate-specific antigen (PSA) compared to the control *in vivo* (Cueto et al. [83]). PSA is a specific PCa marker and used for diagnosis, and its concentration is usually remarkably high in PCa [84]. Therefore, a reduction in PSA levels suggests that coffee oil has defensive actions on the inflammatory status and against prostate hypertrophy. It has been hypothesized that the coffee oil effect was due to 5 alpha-reductase enzyme inhibition (Cueto et al. [83]).

AR has been associated with increased proliferation and altered migratory potential in PCa cells [74]. Coffee diterpenes (kahweol and cafestol) decreased the nuclear AR in AR-positive PCa cells, inhibiting their signals and inducing apoptosis. Coffee diterpenes also caused a reduction in CCR2 and CCR5, without raising their ligands (CCL2 and CCL5) [85].

## 9. Enhancing Insulin Resistance

Fasting serum insulin and insulin resistance are associated with PCa development. Insulin could act as a growth factor and probably raise the androgen entry in prostatic cells by reducing the effect of SHBG. Insulin resistance could raise insulin-like growth factor (IGF), which has mitogenic and antiapoptotic properties, which promote cell proliferation [86]. High circulating insulin also produces proinflammatory responses [4].

**Figure 6:**
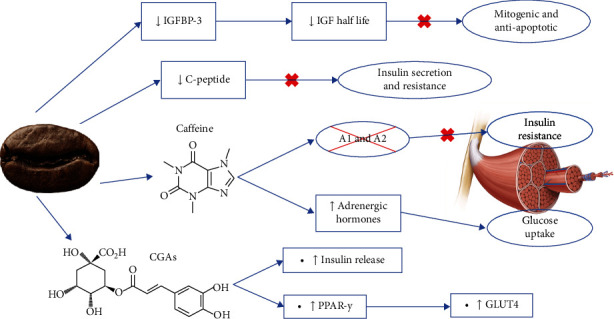
Coffee's mechanism for enhancing insulin resistance.

Besides its antioxidant and anti-inflammatory activities, coffee exerts specific effects that improve glucose and insulin status and is well recognized for preventing and treating type 2 diabetes mellitus (T2DM) [87, 88]. A summary of the mechanism of coffee on glucose metabolism is expressed in Figure 6. These effects are observed both in caffeinated and decaffeinated coffee; however, they might be more significant in decaffeinated coffee ([89, 90], Kumar [91]).

It has been observed that caffeinated coffee consumption reduces IGF-binding protein-3 (IGFBP-3), which extends IGF half-life. C-peptide also decreased due to coffee intake, which is a marker for insulin secretions, indicating lower insulin production and resistance [50]. Moreover, caffeic acid and caffeine reduced IGF-1 receptor expression [63].

It has been observed that incretins raised, and blood glucose reduced after coffee intake. Incretins, such as GLP-1 and GIP, are related to glucose reduction. Additionally, coffee has norharman *β*-carboline, which inhibits *α*-glucosidase activity, which lessens carbohydrate absorption, reducing postprandial glucose levels [89].

Caffeine acts as an antagonist of the A1 and A2 adenosine receptors, which in the skeletal muscle are related to insulin resistance [18, 19, 49]. Caffeine has a synergic effect with adrenergic hormones, which increases glucose intake by tissues. Most of the population quickly metabolizes caffeine; thus, it has acute effects. There is evidence that habitual caffeine intake leads to tolerance to these effects [90].

CGAs are probably responsible for the long-term effects. It has been hypothesized that CGA stimulates insulin release, being a secretagogue. CGA increased the expression of peroxisome proliferator-activated receptor-y (PPAR-y), which is essential in insulin sensitivity. Glucose transporter type 4 (GLUT4) also increased due to CGA, and its expression is stimulated by PPAR-y [92].

## 10. Conclusion

Coffee may reduce the risk of developing PCa through many molecular mechanisms. The ones are antioxidant and anti-inflammatory activities, protection against DNA damage, modulation of transcriptional factors, regulation through microRNA, enhancing steroid metabolism, and enhancing insulin resistance. However, studies vary on serving portions and it is not possible to determine an ideal coffee intake. Therefore, more molecular studies are fundamental to confirm such effects, determine intake recommendations, and assure safety. Coffee may impact PCa through other factors, but more studies are necessary to expand the knowledge on this area and to verify the real extension of the association of coffee consumption and its effects on PCa development.

## Figures and Tables

**Table 1:** Effects of coffee or coffee compound *in vitro*, on cell, animal, or epidemiological models.

Model	Coffee sample/compound	Outcomes	Reference
*In vitro*	Green and roasted *Coffea arabica* and *C. robusta*	(-) Hydroxyl (OH°) radicals DNA damage	[43]
*In vitro*	Coffee phenolics and caffeine metabolites	(-) DNA single-strand breaks	[60]
LNCaP, LNCaP-SF, PC-3, and DU145 cells	Kahweol, cafestol, caffeine, caffeic acid, CGA, and trigonelline	(-) Proliferation and migration of PCa cells (-) AR, CCR2, and CCR5	[85]
Xenograft study of SCID mice		(-) PCa growth	
PC-3 cells	Dark roasted *Coffea arabica*	(-) NF-*κ*B activity and DNA-binding (+) Apoptosis (+) Modulation of gene expression	[8]
PC-3 xenografts in athymic nude mice			
MC3T3-E1 cells	CGA	(-) H_2_O_2_ damage (+) HO-1 and Nrf2 (+) PI3K/Akt	[33]
B16F10 cells	*SCG Coffea arabica*	(-) PI3K/Akt and MAPK	[56]
AML-12 cells	*Coffea arabica* light, medium, city, and French roasts	(+) GSH (+) mRNA related to GSH	[34]
RAW 264.7 cells		(-) TNF-*α* and IL6	
RAW 267.4 cells	SCG	(-) NO production	[26]
CCD-18Co cells	Phenolic compounds from green coffee	(-) ROS production	[40]
HepG2 and KYSE70 cells	Decaffeinated commercial coffee	(+) UGT (-) BaP-induced damage (-) ROS production (+) AhR and Nrf2	[38]
RINm5F and 3T3-L1 cells	CGA	(+) Insulin secretion (+) PPAR-y and GLUT4	(Sanchez et al. 2017)
EA.hy926 cells	Green and light roast *Coffea arabica*	(+) Redox status (+) GSH	[30]
Caco-2 cells	Turkish, filter, and instant coffee	(+) AhR and Nrf2 (+) CYP1A1 expression	[66]
HT29 cells	Blend of green and roasted *Coffea arabica*	(+) Nrf2 transcription and translocation (+) ARE and GST	[29]
HT29 cells	*Coffea arabica*	(+) Nrf2 translocation	[62]
HeLa cells	SCG extracts	(-) ROS level (-) Induced DNA strand breaks	[41]
U-937 cells	SCG	(-) TNF-*α*, IL-6, and IL-10	[48]
SH-SY5Y	Kahweol	(-) ROS and RNS (+) Regulation of PI3K and MAPK pathways	(de [44])
HCT116	Caffeic acid	(+) Regulation of PI3K/Akt	[93]
MCF-7, MDA-MB-231, T47D, and Tam-R cells	Caffeine and caffeic acid	(-) ER abundance (-) IGF1R and pAkt	[63]
C57BL/6 mice	*Coffea arabica* light, medium, city, and French roasts	(-) Liver necrosis (-) IL-6 (-) TNF-*α* (+) NF-*κ*B pathway	[28]
C57BL/6 mice	Low-molecular-weight from regular and decaffeinated *Coffea canephora*	(-) MicroRNA-124-3p (+) mRNA related to GPX Decaffeinated coffee had no effect	[37]
C57BL/6 mice	Regular and decaffeinated coffee	(+) Autophagic flux (+) Lipidation of LC3B (-) p62/SQSTM1 (-) mTORC1 (+) Deacetylation of cellular proteins	[52]
HtgUGT1A mice	Caffeic acid	(+) UGT (-) ROS	[39]
Sprague Dawley rats	*Coffea arabica* oil	(-) PSA	(Cueto et al. 2016)
Him-OFA rats	Regular and decaffeinated *Coffea arabica*	(-) Hepatic foci frequency (-) Aflatoxin DNA damage (+) UGT	[59]
Wistar rats	A medium roast of *Coffea arabica*	(+) SOD, CAT, and GPx	[36]
Wistar rats	Green *Coffea arabica*	(-) Lipid peroxidation (+) GSH, SOD, CAT, and GR	[35]
Wistar rats	Green *Coffea canephora* and caffeine	(-) Carrageenan-induced paw oedema	(Pergolizzi et al. 2018)
Wistar rats	Green *Coffea arabica*	(-) IL-1*β*, TNF-*α*, and Nos2 (-) Interstitial inflammation index	[47]
Humanized SHBG transgenic mice	Caffeine	(+) Hepatic SHBG production (-) Akt phosphorylation	[77]
Cross-sectional clinical trial	Caffeinated beverages	Coffee consumption positively associated with SHBG concentration	[78]
Prospective clinical trial	Caffeinated and decaffeinated coffee	(-) CRP, IL-6, and TNFR-2 (+) Adiponectin and SHBG (-) Estrone (+) Testosterone (-) C-peptide and IGFBP-3	[50]
Prospective clinical trial	Coffee intake	(-) Urinary 8-OHdG (-) Ferritin	[61]
Prospective clinical trial	Coffee intake	(+) AhR (+) CYP1A1/A2	[65]
Intervention clinical trial	Green and roasted *Coffea arabica* blend	(-) Spontaneous DNA strand breaks	([57]; 2014; 2011)
Intervention clinical trial	*Coffea arabica*	(-) DNA strand breaks (+) Nrf2 signaling	[31]
Intervention clinical trial	Caffeinated and decaffeinated coffee	(+) Insulin sensitivity (+) GLP-1 and GIP	[89]

Legend: (-) = reduction/inhibition; (+) = increase/activation/improvement; CGA = chlorogenic acid; SCG = spent coffee ground.

## Data Availability

The data used to support the findings of this study are included within the article.
